# Effect of Arbuscular Mycorrhizal Fungi on the Growth and Cadmium Uptake of *Paspalum notatum* and *Lolium perenne*

**DOI:** 10.3390/jof12020099

**Published:** 2026-01-30

**Authors:** Chao Wang, Peiyin Li, Ao Yuan, Zhiwei Bian, Huiping Song, Zhengjun Feng

**Affiliations:** 1 Institute of Resources and Environmental Engineering, Shanxi University, Taiyuan 030006, China; wangchao20251218@126.com (C.W.); lipeiyin11@126.com (P.L.); yuanao0713@163.com (A.Y.); 2 Shanxi Shanshui Cement Company Limited, Taiyuan 030006, China; 3 China-Mongolia Belt and Road Joint Laboratory of Mineral Processing Technology, Inner Mongolia Academy of Science and Technology, Hohhot 010000, China; bianzhiwei@imast.ac.cn

**Keywords:** heavy metal stress, mycorrhizal dependence, plant response, plant fungus interaction, bioremediation potential, growth stability

## Abstract

To investigate the regulatory mechanisms of arbuscular mycorrhizal fungi (AMF) on the growth, cadmium (Cd) uptake and translocation of plants with distinct mycorrhizal dependency (MD), a pot experiment was conducted using *Paspalum notatum* (high MD) and *Lolium perenne* (low MD) under two Cd gradients (5 mg·kg^−1^, 50 mg·kg^−1^) with AMF-inoculated/non-inoculated treatments, with 0 mg·kg^−1^ set as the control group. AMF significantly enhanced the dry weight and colonization rate of both plant species, and the MD of *Paspalum notatum* remained consistently higher. The growth-promoting effect of AMF (quantified by MD) exceeded the toxicity induced by Cd stress, thereby mitigating growth inhibition by promoting hyphal growth in the rhizosphere. AMF improved the root bioconcentration factor (BCF) and total Cd extraction capacity of the plants, which was correlated with the plants’ inherent Cd absorption capacity but not with MD. AMF exerted species-specific regulatory effects on the translocation factor (TF): the TF of *Paspalum notatum* increased after inoculation, while that of *Lolium perenne* decreased.

## 1. Introduction

As heavy metal pollution became a global issue, cadmium (Cd) was regarded as one of the most toxic pollutants and posed severe environmental and health risks [1,2]. It was also considered one of the most serious soil pollutants in China. Cd can persist and accumulate in soils over long periods, posing substantial risks to plant health and ecosystem stability [3]. Phytoremediation of Cd-contaminated soils and risk-controllable cultivation on contaminated soils are two common approaches for addressing Cd pollution. Therefore, from the perspective of human health protection and plant normal growth, the research on plant Cd uptake and translocation process and influencing factors was of great help to control Cd pollution and mitigate its harm, which had aroused widespread attention in relevant research areas for a long time.

Arbuscular mycorrhizal fungi (AMF) form symbiotic relationships with most plants [4,5]. AMF influence plant Cd accumulation and tolerance through multiple mechanisms. AMF enhance nutrient and water uptake in host plants by expanding the root surface area, thereby promoting plant growth [6,7]. AMF also increases the concentration of permeation-regulating substances, enhances antioxidant capacity, stabilizes cell membranes, and alleviates Cd-induced oxidative stress in host plants [8]. AMF have demonstrated significant potential in mitigating Cd toxicity and restoring soil health [9]. In Cd-contaminated environments, AMF reduce toxicity through multiple mechanisms. They alter rhizosphere chemistry by secreting Cd-binding proteins and chelating compounds, thereby promoting Cd immobilization [10]. AMF can upregulate the expression of genes associated with heavy metal chelation in plant roots, promoting the synthesis of more plant chelating peptides and metallothioneins. These substances bind with Cd ions to form low-toxicity or non-toxic complexes [11]. To some degree, AMF enhanced plant tolerance to heavy metals; therefore, the study of AMF inoculation on plant heavy metal uptake and translocation, as well as growth changes, has always been a hot research topic. Previous studies have shown that AMF-mediated regulation of heavy metal dynamics is shaped by multiple factors, including soil properties, metal concentrations, AMF species, and host plant identity [12].

When plants are used for the remediation of heavy metal-contaminated soils, the choice of plant species and AMF strains strongly affects remediation efficiency by altering plant metal uptake. For example, in the remediation of Cd-contaminated soils, *Rhizophagus irregularis* promotes the uptake of heavy metals by *Helianthus annuus L.*, whereas inoculation of *Zea mays* L. with *Glomus constrictum* elicited a negative effect [13]. Some plants increased heavy metal tolerance and decreased heavy metal accumulation in aboveground parts with AMF, whereas other plants increased heavy metal uptake and translocation to shoots with AMF [14]. These contrasting outcomes were largely attributable to interspecific differences in root morphology, root exudation patterns, metal transporter regulation, and intrinsic detoxification mechanisms. All these factors shaped how AMF symbiosis influenced plant heavy metal uptake and translocation [15].

Mycorrhizal dependence (MD) is the degree to which plants reach their maximum growth or yield under given soil fertility levels as a result of their symbiosis with AMF [16]. Plants from different functional groups (herbs, woody plants, legumes, etc.) often responded differently to AMF inoculation. MD also varied with inoculation type [17], and it was significantly different for plants of different species and functional groups [18]. Previous studies primarily focused on the relationship between MD and plant growth (such as biomass) [19], whereas few studies investigated changes in plant heavy metal uptake associated with MD [20].

*Paspalum notatum*, a warm-season C_4_ grass with a well-developed root system, typically showed high MD under natural conditions [21]. In contrast, *Lolium perenne*, a cool-season C_3_ species, exhibited moderate-to-low MD due to its efficient intrinsic nutrient uptake capacity [22]. Although both species benefit from AMF under nutrient-deficient conditions, the MD of *Paspalum notatum* consistently exceeds that of *Lolium perenne*. Despite these clear interspecific differences, the relationship between MD and plant heavy metal uptake capacity remained insufficiently understood. However, it remained unclear whether variation in MD was accompanied by corresponding differences in heavy metal uptake capacity.

This study hypothesized that *Paspalum notatum*, which exhibited higher MD, would be more strongly affected by AMF inoculation. In particular, AMF was expected to exert greater effects on Cd uptake and translocation in *Paspalum notatum*, whereas *Lolium perenne* was expected to show the opposite response. We selected two grass species, *Paspalum notatum* and *Lolium perenne*, which differed markedly in MD, Cd uptake capacity, and responsiveness to AMF. A pot experiment was set up to explore the effects of AMF on heavy metal uptake and growth performance of the two species under two different heavy metal Cd contamination gradients.

## 2. Materials and Methods

### 2.1. Experimental Materials

This study employed two gramineous species, *Lolium perenne* and *Paspalum notatum*. *Lolium perenne*, a perennial cool-season grass, was obtained from Zhongjiang Seed Industry Co., Ltd. (Nanjing, China), while *Paspalum notatum*, a perennial warm-season species, was supplied by Guangzhou Tianye Feng Ecological Garden Co., Ltd. (Guangzhou, China).

The AMF inoculum used in the experiment consisted of *Rhizophagus irregularis* (commercial powdered formulation; Symbiom, Czech Republic) with a spore density of 90 spores·g^−1^. *Rhizophagus irregularis* can colonize over 90% of terrestrial plants, making it suitable for diverse cultivation scenarios. It exhibits rapid infection rates and is compatible with most crops, including corn, wheat, and vegetables [23].

Experimental soil was collected from Wuxiang County, Shanxi Province, and its baseline physicochemical properties were analyzed following the “Soil Physicochemical Property Determination” standard (Table 1).

### 2.2. Experimental Design

A pot experiment was conducted using sterilized plastic containers (12.5 × 17 × 16 cm), each filled with 2300 g of soil. Three Cd contamination levels were established (0, 5, and 50 mg·kg^−1^), and for each level, two treatments were applied: non-mycorrhizal (NM) and AMF-inoculated (AM). Each treatment contained five replicates, resulting in a total of 60 pots, with 30 pots assigned to *Lolium perenne* and 30 to *Paspalum notatum*.

Seeds of both species were surface-sterilized with 10% H_2_O_2_ for 10 min, rinsed 5–6 times with ultrapure water, and air-dried prior to sowing. For each pot, 1.50 g of seeds was sown at a depth of 2 cm. AMF treatment pots received 5 g of *Rhizophagus irregularis* inoculum, which was evenly distributed at approximately two-thirds of the soil depth, whereas NM pots received 5 g of autoclaved fungal spores to maintain consistent soil conditions.

All pots were maintained in a controlled greenhouse environment at 26–30 °C, with 60% (±5%) relative humidity and a 14-h photoperiod. Plants were irrigated with ultrapure water during early establishment. 10 days after sowing, 100 mL of phosphorus-depleted Hoagland’s nutrient solution was supplied to each pot at 7-day intervals for the duration of the experiment.

### 2.3. Sample Collection and Biological Indicators

After 60 days of growth, plants of *Lolium perenne* and *Paspalum notatum* were harvested. Roots were gently shaken to remove loosely adhering soil, then rinsed thoroughly with ultrapure water and air-dried to eliminate surface moisture prior to subsequent analyses. Plant height was measured using a flexible ruler, with multiple measurements taken per pot and averaged for analysis. Fresh biomass of shoots and roots was determined using an analytical balance, and values represent the mean of repeated measurements.

### 2.4. Determination of Cd Concentration

Soil Cd concentrations were analyzed following the procedures described in “Soil Agronomic Chemical Analysis” [24]. For Cd determination in plant tissues, 0.1 g of dried and ground sample was placed into a polytetrafluoroethylene (PTFE) digestion vessel, moistened with several drops of ultrapure water, and treated with an acid digestion solution (3 mL hydrochloric acid, 1 mL nitric acid, and 1 mL hydrofluoric acid). Samples were digested using a standardized microwave digestion program, after which the digest was transferred to a water bath for acid evaporation, cooled, and diluted to 25 mL with ultrapure water in a volumetric flask. Cd concentrations were quantified using inductively coupled plasma optical emission spectrometry (ICAP 6000, Thermo Fisher Scientific, Waltham, MA, USA).

### 2.5. Determination of Mycorrhizal Colonization Rate

When harvesting plants, several plant roots were sampled, and the staining method was adopted to determine the mycorrhizal infection rate [25]. The specific procedure is as follows:

(1) Root sampling: Place 0.5–1.0 g of 1 cm root segments into a perforated film canister for AM colonization rate assessment.
(2) Root digestion: Immerse root segments in 10% KOH (*m*/*v*) (ensuring full coverage), incubate in a 90 °C water bath for ~1 h until colorless, then rinse thoroughly with running water.
(3) Acidification: Soak in 2% HCl (*v*/*v*) for 5 min to neutralize and soften the roots.
(4) Staining: Discard HCl, add 0.05% (*m*/*v*) acid fuchsin solution (prepared with glycerol lactate solution), stain in a 90 °C water bath for ~30 min, then rinse with water until the eluate is nearly colorless.
(5) Decolorization: Transfer to a Petri dish with glycerol lactate solution (lactic acid/glycerol/water = 1:1:1, *v*/*v*/*v*) and decolorize for at least 8 h.

Mycorrhizal colonization rate (%) was calculated as:

Colonization rate = Σ(0% × N_0_ + 10% × N_1_ + 30% × N_2_ + 50% × N_3_ + 70% × N_4_ + 90% × N_5_)/N(total)

where N_i_ denotes the number of root segments in infection class i, and N(total) is the total number of root segments observed.

### 2.6. Mycorrhizal Dependency

Mycorrhizal dependency (MD) quantifies the extent to which a plant relies on AMF to achieve maximum growth under defined soil fertility conditions [26]. The following formula was used to calculate the MD of maize varieties [27]:

$$MD=\frac{\mathrm{TB}(\mathrm{Myc})-\mathrm{TB}(\mathrm{NonMyc})}{\mathrm{TB}(\mathrm{Myc})}\times 100$$

MD: mycorrhizal dependency (%), TB (Myc): the total biomass of inoculated plants, and TB (NonMyc): the total biomass of uninoculated plants.

### 2.7. Bioconcentration Factor

Heavy metals bioconcentration factor (BCF) of plants is one of the important indices to evaluate plants’ ability to adsorb and enrich heavy metals in soil or water [28]. BCF is defined as follows:

$$BCF=\frac{The\;content\;of\;a\;certain\;heavy\;metal\;in\;plants}{The\;content\;of\;corresponding\;heavy\;metals\;in\;soil\;or\;water}$$

Usually, the heavy metal concentration in the plant is represented by the mass concentration (mg·kg^−1^), and the heavy metal content in the soil is also represented by mass concentration (mg·kg^−1^) [29]. The greater BCF means that the plant can absorb the heavy metal more efficiently. When the BCF is greater than 1, it indicates that the plant can absorb more heavy metals from the environment. When BCF is less than 1, it indicates that the plant’s ability to absorb heavy metals is weaker, possibly an excluder of heavy metals [30].

### 2.8. Translocation Factor

The translocation factor (TF) is used to assess a plant’s ability to transfer heavy metals from roots to aboveground tissues [31]. TF is calculated as follows:

$$TF=\frac{Heavy\;metal\;concentrations\;in\;the\;aboveground\;parts\;of\;plants}{Heavy\;metal\;concentrations\;in\;plant\;roots}$$

A TF greater than 1 indicates efficient root-to-shoot translocation and is often used as a criterion for identifying hyperaccumulator species. In contrast, TF values less than or equal to 1 reflect limited metal mobility within the plant [30].

### 2.9. Statistical Analysis

A two-way ANOVA was conducted to determine the effects of AM inoculation and Cd exposure on mycorrhizal dependency, mycorrhizal colonization rate, bioconcentration factor, and translocation factor. The post hoc analysis was performed using the Duncan multiple-comparison test. Statistical analyses were conducted using IBM SPSS Statistics 23.0 (IBM Corp., Armonk, NY, USA). Data are presented as mean ± standard deviation (n = 5), and differences were considered statistically significant at *p* < 0.05.

## 3. Results

### 3.1. Comparison of Heavy Metal Cd Accumulation Between Two Plants

**Figure 1:**
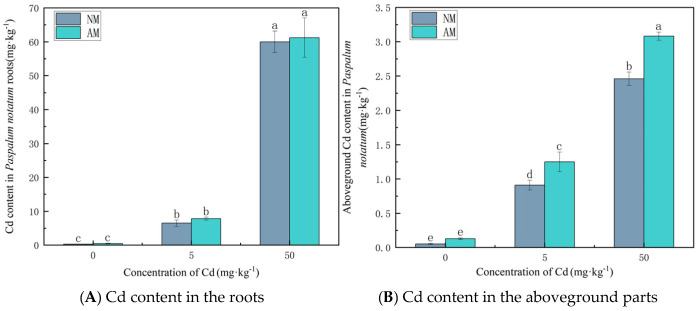
Cd concentrations in the root (**A**) and the aboveground parts (**B**) of *Paspalum notatum* under different treatments. Note: Values with similar letters do not differ statistically according to the Duncan test at *p* < 0.05. NM denotes the non-mycorrhizal (non-inoculated) treatment, whereas AM denotes the arbuscular mycorrhizal (AM) fungal inoculation treatment.

As shown in Figure 1, Cd concentrations in the roots and shoots of *Paspalum notatum* increased progressively with rising soil Cd levels. AMF inoculation increased Cd uptake in the roots, but the increase was not statistically significant. In contrast, AMF markedly promoted Cd accumulation in the shoots. At soil Cd concentrations of 5 and 50 mg·kg^−1^, AMF inoculation increased shoot Cd concentrations by 37.08% and 24.84%, respectively (*p* < 0.05).

**Figure 2:**
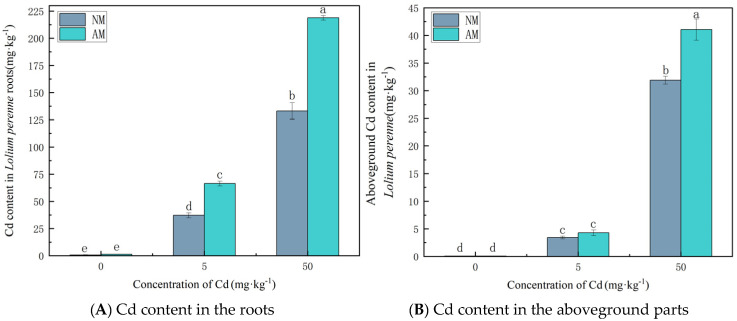
Cd concentrations in the roots (**A**) and the aboveground parts (**B**) of *Lolium perenne* under different treatment conditions. Note: Values with similar letters do not differ statistically according to the Duncan test at *p* < 0.05. NM denotes the non-mycorrhizal (non-inoculated) treatment, whereas AM denotes the arbuscular mycorrhizal (AM) fungal inoculation treatment.

As indicated by Figure 2, Cd concentrations in both roots and shoots of *Lolium perenne* increase with increasing soil Cd levels. Across all Cd treatments, AMF inoculation significantly enhanced Cd accumulation in both tissues, with roots exhibiting much higher Cd levels than shoots. Compared with the NM treatment, AMF inoculation increased root Cd concentrations by 79.46%, 79.05%, and 64.34% at 0, 5, and 50 mg·kg^−1^ Cd, respectively. Shoot Cd concentrations were also elevated under AMF inoculation, with increases of 25.68%, 24.85%, and 28.68% at the corresponding Cd levels.

### 3.2. Comparison of the Cd BCF in Lolium perenne and Paspalum notatum

**Figure 3:**
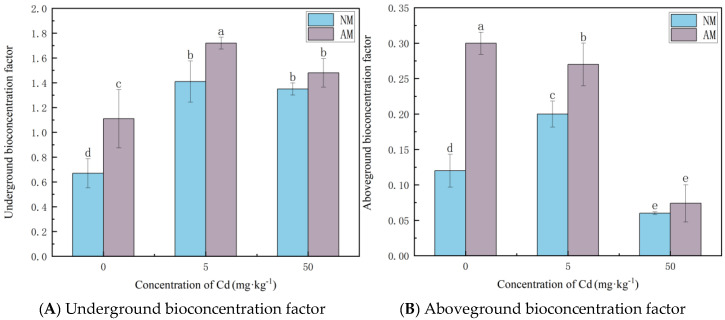
The effect of AMF on the BCF of Cd in the root (**A**) and the aboveground parts (**B**) of *Paspalum notatum.* Note: Values with similar letters do not differ statistically according to the Duncan test at *p* < 0.05. NM denotes the non-mycorrhizal (non-inoculated) treatment, whereas AM denotes the arbuscular mycorrhizal (AM) fungal inoculation treatment.

As indicated by Figure 3, the root BCF of *Paspalum notatum* increased with Cd level and peaked at 5 mg·kg^−1^, indicating a strong Cd accumulation capacity in roots. In contrast, shoot BCF values remained far below 1 across all treatments, reflecting limited Cd accumulation in aboveground tissues. At all Cd levels, root BCF values were substantially higher than shoot BCF values. Moreover, AMF inoculation consistently increased BCF in both root and shoot BCF compared with the NM treatment.

**Figure 4:**
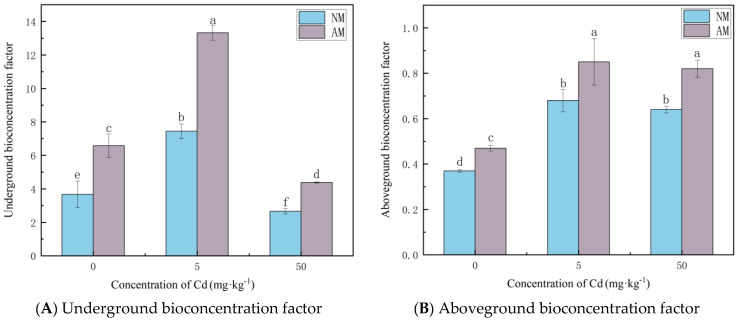
The effect of AMF on the BCF of Cd in the root (**A**) and the aboveground parts (**B**) of *Lolium perenne.* Note: Values with similar letters do not differ statistically according to the Duncan test at *p* < 0.05. NM denotes the non-mycorrhizal (non-inoculated) treatment, whereas AM denotes the arbuscular mycorrhizal (AM) fungal inoculation treatment.

As indicated by Figure 4, the root BCF of *Lolium perenne* increased with increasing Cd levels and peaked at 5 mg·kg^−1^, but declined at 50 mg·kg^−1^, indicating reduced Cd accumulation efficiency under high Cd stress. AMF inoculation significantly increased BCF in both roots and shoots across all Cd treatments. Similar to *Paspalum notatum*, root BCF values were consistently higher than shoot BCF values, and root BCF remained greater than 1 under all treatments.

### 3.3. Comparison of the Cd TF in Lolium perenne and Paspalum notatum

**Figure 5:**
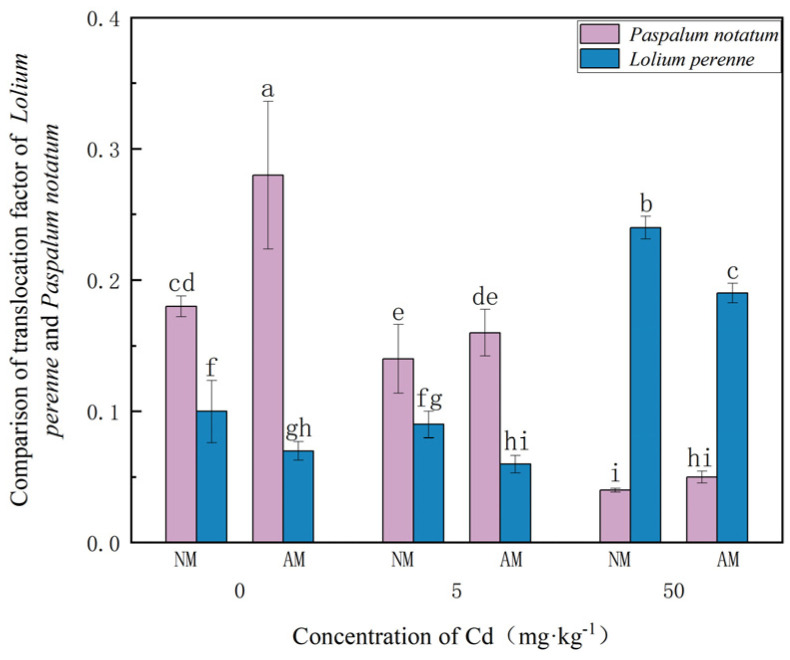
Comparison of Cd TF between *Paspalum notatum* and *Lolium perenne* at different Cd concentrations. Note: Values with similar letters do not differ statistically according to the Duncan test at *p* < 0.05. NM denotes the non-mycorrhizal (non-inoculated) treatment, whereas AM denotes the arbuscular mycorrhizal (AM) fungal inoculation treatment.

As shown in Figure 5. TF values for both species under different Cd treatments revealed that, in *Paspalum notatum*, TF values remained well below 1 across all treatments, indicating limited Cd mobility to shoots. AMF inoculation increased TF at 0 mg·kg^−1^ Cd, with a 55.6% rise, but TF declined progressively as Cd levels increased.

In *Lolium perenne*, TF values were extremely low (<0.3) under all treatments. TF increased slightly with increasing Cd levels, and significant differences were observed between Cd ≤ 5 mg·kg^−1^ and 50 mg·kg^−1^ treatments. Unlike *Paspalum notatum*, AMF inoculation consistently reduced TF in *Lolium perenne*, with the greatest reduction (21.7%) occurring at 50 mg·kg^−1^ Cd. Collectively, these results demonstrate species-specific differences in AMF-mediated Cd translocation.

### 3.4. The Effect of AMF on Paspalum notatum and Lolium perenne

As shown in Table 2, AMF inoculation increased dry weight in both *Paspalum notatum* and *Lolium perenne* across all Cd treatments. For *Paspalum notatum*, dry weight showed an initial increase followed by a decrease as Cd levels increased, whereas dry weight in *Lolium perenne* gradually decreased. Consistent with previous observations, *Paspalum notatum* exhibited higher MD values than *Lolium perenne* across all treatments.

**Figure 6:**
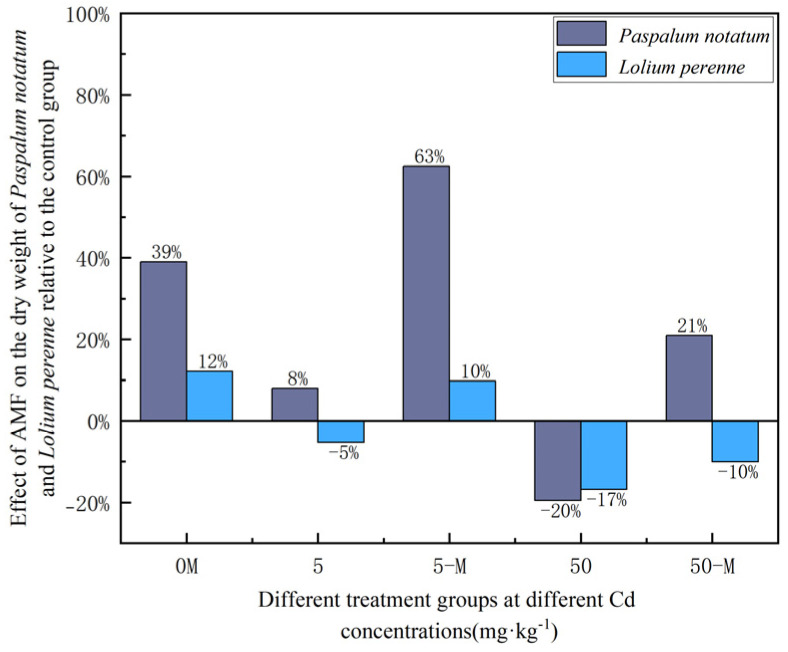
The effect of AMF on the relative dry weight of *Paspalum notatum* and *Lolium perenne*.

As shown in Figure 6, relative dry weight patterns indicated that, in *Paspalum notatum*, dry weight decreased in the NM treatment at 50 mg·kg^−1^ Cd, whereas AMF inoculation resulted in increased dry weight at the same Cd level. In *Lolium perenne*, a similar pattern was observed at 5 mg·kg^−1^ Cd, where NM plants showed reduced dry weight, but AM plants exhibited increased dry weight. Although dry weight declined in both species at 50 mg·kg^−1^ Cd, the reduction was consistently smaller in AM plants than in NM plants. These results illustrate the protective role of AMF in mitigating Cd-induced growth inhibition.

Mycorrhizal colonization data are shown in Table 3. AMF inoculation resulted in significantly higher colonization rates in both *Paspalum notatum* and *Lolium perenne* compared with the NM treatment. In *Paspalum notatum*, colonization exhibited a unimodal response to increasing Cd levels, increasing initially and then declining at high Cd levels. In contrast, colonization in *Lolium perenne* decreased progressively with increasing Cd exposure.

## 4. Discussion

The MD values of *Paspalum notatum* ranged from 28% to 34%, while those of *Lolium perenne* ranged from 7% and 14%, showing significant interspecific differences. According to the literature, reported MD values varied widely among plant species, ranging from approximately 10% to 90%. For example, MD values of *Bouteloua curtipendula* (87%), *Festuca arundinacea* (56%), *Mimosa biuncifera* (44%), *Setaria glauca* (17%), and *Triticum aestivum* (14%) were reported previously [32]. MD values were likely influenced by plant species, AMF species, and cultivation conditions. In this study, the MD value of *Lolium perenne* was consistent with the previously reported value (16%) [32]. The MD values obtained for *Paspalum notatum* and *Lolium perenne* in this experiment fell within a reasonable range, with *Paspalum notatum* showing a higher MD than *Lolium perenne*. Notably, consistent with the findings of Solís-Rodríguez, U. et al. [33], the interspecific difference in MD remained stable under heavy metal stress.

Cd exerted growth-inhibitory effects on both *Paspalum notatum* and *Lolium perenne*, and this toxicity was primarily indicated by changes in plant dry weight, a key indicator of growth. However, the two species responded differently to Cd stress. *Paspalum notatum* showed an increasing trend in dry weight under low Cd stress (5 mg·kg^−1^), exhibiting a certain degree of Cd tolerance and growth-promoting effect. In contrast, *Lolium perenne* was significantly inhibited across all tested Cd concentration gradients, with its dry weight continuously decreasing as soil Cd concentration increased.

AMF counteracted the negative impacts of heavy metals primarily through multiple synergistic mechanisms. First, AMF expanded the root-soil contact area, enhanced the absorption efficiency of plants for nutrients such as trace elements and minerals (e.g., phosphorus, potassium), and improved water use efficiency. These effects increased key growth traits such as plant height and biomass, thereby alleviating Cd-induced growth inhibition [34]. Second, AMF regulated the expression of heavy metal transporters in plant roots to inhibit the absorption and translocation of Cd^2+^. Meanwhile, AMF enhanced the activities of antioxidant enzymes, such as superoxide dismutase (SOD) and peroxidase (POD), thereby improving plant physiological tolerance to Cd stress. In addition, AMF hyphae adsorbed and immobilized Cd in the soil and released exudates (e.g., organic acids) to chelate free Cd. AMF also altered the distribution of bioavailable Cd fractions, for example, by reducing the proportion of exchangeable Cd, thus decreasing Cd uptake at the soil–root interface and mitigating toxicity [35].

Notably, previous studies reported that AMF inoculation significantly reduced the Cd^2+^ uptake efficiency of *Medicago truncatula* [36], thereby alleviating Cd-induced damage to plant physiological metabolism and photosynthesis. However, this mechanism was not consistent with the observations of the present study. Based on the growth responses of the two species and the observed AMF effects in this study, it was inferred that AMF counteracted Cd toxicity in *Paspalum notatum* and *Lolium perenne* mainly by enhancing nutrient uptake and water use efficiency, thereby improving overall plant growth performance.

There were significant interspecific differences in the Cd absorption capacity between *Paspalum notatum* and *Lolium perenne*. Specifically, *Paspalum notatum* had a lower total Cd absorption, while *Lolium perenne* exhibited a higher Cd uptake [37], which was closely related to the differences in their inherent physiological characteristics and root absorption mechanisms. Notably, after inoculation with AMF, the Cd absorption of both plant species increased to varying degrees, indicating that AMF exerted a significant promoting effect on plant heavy metal absorption. Regarding the characteristics of Cd translocation and distribution, AMF inoculation induced diametrically opposite regulatory effects on the TF of the two plant species: the TF value of *Paspalum notatum* was significantly higher than that of the non-inoculated treatment, implying that AMF enhanced the translocation efficiency of Cd from its roots to shoots; in contrast, the TF value of *Lolium perenne* showed a significant decreasing trend. This difference indicated that AMF effectively inhibited the migration and translocation of heavy metals from roots to shoots by enhancing the Cd adsorption and accumulation capacity of *Lolium perenne* roots, thereby reducing the risk of Cd accumulation in aboveground tissues [38].

Taking the Cd absorption capacity of the roots of the two plant species as a specific observation index, after inoculation with AMF, the root Cd absorption of both species increased to varying degrees. For Paspalum notatum, root Cd absorption increased by 56.67%, 20.39%, and 2.03% under soil Cd concentrations of 0 mg·kg^−1^, 5 mg·kg^−1^, and 50 mg·kg^−1^, respectively, compared with the non-inoculated treatment. In contrast, Lolium perenne exhibited much higher increase rates of root Cd absorption under the same Cd gradients, reaching 79.46%, 79.05%, and 64.34%, respectively. This observation was inconsistent with the initial hypothesis of this study—the increase in root Cd absorption of *Lolium perenne* (low MD: 7%~14%) after AMF inoculation was significantly higher than that of *Paspalum notatum* (high MD: 28%~34%). These results suggested that the AMF-promoted Cd uptake in roots was not necessarily related to plant MD.

## 5. Conclusions

AMF had positive effects on both plants, and the positive effects were influenced by MD. Heavy metals had negative effects on the plants, and the negative effects could counterbalance the positive effects of AMF.

AMF could promote the development of the plant root system, and then improve the bioaccumulation efficiency of plant roots to heavy metals. Although AMF could facilitate the uptake of heavy metals to the aboveground parts to a certain degree, the negative impact caused by the additional heavy metals was completely eliminated by the positive effects of AMF.

The effects of AMF on Cd uptake were related to plant species, but did not seem to depend on the MD, but rather the inherent Cd absorption capacity of the plants themselves. In plants with weak inherent Cd absorption capacity, AMF tended to enhance root-to-shoot TF. Meanwhile, for plants with strong inherent Cd absorption capacity, the AMF tended to inhibit root-to-shoot TF, but this may also be due to a reduction in uptake capacity.

## Figures and Tables

**Table 1:** Soil physical-chemical properties.

pH	Soil Organic Matter (g·kg^−1^)	Total Nitrogen (g·kg^−1^)	Available Phosphorus (mg·kg^−1^)	Total Cadmium Content (mg·kg^−1^)	Available Potassium (ppm)	Cation Exchange Capacity (cmol/kg)
8.57	32.33	0.7	15.61	0.58	17.30	32.24

**Table 2:** The effect of AMF on the dry weight of the two plants and the MD values of the two plants under different Cd concentrations. Different letters in the table indicate significant differences (*p* < 0.05).

Cd Addition (mg·kg^−1^)	Plant Species	Dry Weight (g)		MD (%)
		NM	AM	
0	*Paspalum notatum*	2.00 ± 0.05 d	2.78 ± 0.05 d	28.06
	*Lolium perenne*	8.38 ± 0.20 a	9.40 ± 0.36 a	10.85
5	*Paspalum notatum*	2.16 ± 0.05 d	3.25 ± 0.04 c	33.54
	*Lolium perenne*	7.94 ± 0.19 b	9.20 ± 0.48 a	13.70
50	*Paspalum notatum*	1.61 ± 0.04 e	2.42 ± 0.02 e	33.47
	*Lolium perenne*	6.98 ± 0.23 c	7.54 ± 0.20 b	7.43

**Table 3:** The mycorrhizal colonization rate of *Paspalum notatum* and *Lolium perenne*. Different letters in the table indicate significant differences (*p* < 0.05).

Plant Species	Cd Addition (mg·kg^−1^)	Mycorrhizal Colonization Rate (%)	
		NM	AM
*Paspalum notatum*	0	10.97 ± 2.10 c	39.07 ± 5.90 b
	5	13.92 ± 3.72 c	53.37 ± 3.44 a
	50	8.96 ± 1.23 c	30.86 ± 3.03 b
*Lolium perenne*	0	10.44 ± 1.50 c	56.71 ± 5.98 a
	5	9.28 ± 0.55 c	52.64 ± 2.44 a
	50	8.07 ± 1.11 c	42.68 ± 7.34 ab

## Data Availability

The original contributions presented in this study are included in the article. Further inquiries can be directed to the corresponding authors.
